# Heterogeneous Pancreatic Stellate Cells Are Powerful Contributors to the Malignant Progression of Pancreatic Cancer

**DOI:** 10.3389/fcell.2021.783617

**Published:** 2021-12-20

**Authors:** Zhilin Zhang, Huan Zhang, Tian Liu, Tian Chen, Daorong Wang, Dong Tang

**Affiliations:** ^1^ Clinical Medical College, Yangzhou University, Yangzhou, China; ^2^ Department of General Surgery, Northern Jiangsu People’s Hospital, Clinical Medical College, Institute of General Surgery, Yangzhou University, Yangzhou, China

**Keywords:** inflammation, fibrosis, pancreatic stellate cells, pancreatic neoplasms, antineoplastic protocols

## Abstract

Pancreatic cancer is associated with highly malignant tumors and poor prognosis due to strong therapeutic resistance. Accumulating evidence shows that activated pancreatic stellate cells (PSC) play an important role in the malignant progression of pancreatic cancer. In recent years, the rapid development of single-cell sequencing technology has facilitated the analysis of PSC population heterogeneity, allowing for the elucidation of the relationship between different subsets of cells with tumor development and therapeutic resistance. Researchers have identified two spatially separated, functionally complementary, and reversible subtypes, namely myofibroblastic and inflammatory PSC. Myofibroblastic PSC produce large amounts of pro-fibroproliferative collagen fibers, whereas inflammatory PSC express large amounts of inflammatory cytokines. These distinct cell subtypes cooperate to create a microenvironment suitable for cancer cell survival. Therefore, further understanding of the differentiation of PSC and their distinct functions will provide insight into more effective treatment options for pancreatic cancer patients.

## 1 Introduction

Pancreatic cancer is among the most deadly forms of cancer, with a mortality-to-incidence ratio of 0.82 in 2020 and a 5 year survival rate of about 9% (Mizrahi et al., 2020). Associated symptoms typically go undetected until the advanced stages of the disease, resulting in great difficulty in early diagnosis (Kamisawa et al., 2016). Although surgical resection remains the preferred treatment option for pancreatic cancer patients, it is not effective in cases involving distant metastases. Interstitial cells, especially pancreatic stellate cells (PSC), play an important role in the malignant progression and treatment resistance observed in pancreatic cancer patients. PSC promote the fibrosis and inflammatory response of the tumor microenvironment by producing a large number of collagen fibers, exosomes, and soluble factors, which provide the basis for the proliferation, migration, and immune escape of pancreatic cancer cells (Wang et al., 2020), (Wang et al., 2017). More importantly, PSC play an extremely critical role in promoting the epithelial-mesenchymal transition (EMT) of cancer cells and enhancing cancer cell stemness. PSC promote EMT in pancreatic cancer cells by secreting exosomal microRNA-21 (Ma et al., 2020). It was shown that PSC also induce cancer stem cell-like properties in cancer cells through the secretion of osteopontin (Cao et al., 2019). Moreover, PSC also promote EMT in cancer cells by secreting hepatocyte growth factor (Xu et al., 2020), and the hepatocyte growth factor also promotes the expression of cancer stem cell pluripotency markers in cancer cells (Yan et al., 2018).

With the rapid development of single-cell sequencing technology, the heterogeneity of PSC populations has been recently investigated and attracted the attention of researchers, as this may provide a potential therapeutic route to targeting the tumor microenvironment (Pothula et al., 2020). Recently, researchers have identified two spatially separated, functionally complementary, and reversible subtypes of PSC in pancreatic cancer tissues, namely myofibroblastic and inflammatory PSC. Myofibroblastic PSC, characterized by elevated a-smooth muscle actin (*α*-SMA) expression, produce large amounts of pro-fibroproliferative collagen fibers, whereas inflammatory PSC express large amounts of inflammatory cytokines. Importantly, these two subtypes of PSC have been shown to operate synergistically to promote the progression of pancreatic cancer (Öhlund et al., 2017). Other PSC subtypes have also been identified. Cluster of differentiation (CD) 10-positive PSC assists in cancer cell invasion (Ikenaga et al., 2010). Bcl2‐associated athanogene- or fibroblast activation protein (FAP)-positive PSC not only enhance cancer cell migration but also promote fibrosis (Yuan et al., 2019), (Feig et al., 2013). There are even examples of differentiation among tumor suppressor subtypes, such as subsets expressing CD271 or Meflin (Nielsen et al., 2018), (Mizutani et al., 2019). However, improving patient survival requires a more detailed understanding of the mechanisms underlying PSC differentiation and their role in tumor malignancy. In the present review, we discuss the differentiation mechanism and cancer-promoting functions of myofibroblastic and inflammatory PSC. Moreover, we propose a more effective approach to manage treatment resistance in pancreatic cancer, at the level of PSC heterogeneity.

## Article Category: Review

### Differentiation Mechanism of Myofibroblastic and Inflammatory PSC

Cancer-associated fibroblasts (CAF) that are induced by cancer cells are mesenchymal-derived cells and play an active role in promoting pancreatic cancer progression. CAF in the pancreas are highly heterogeneous, and researchers have recently identified cancer-promoting inflammatory CAF and myofibroblastic CAF as well as cancer-suppressing CAF that exert antigen-presenting ability (Hosein et al., 2020). Different groups of CAF have very different functions, for example, inflammatory CAF mainly lead to the inflammatory response of cancer stroma, while myofibroblastic CAF mainly lead to stroma fibrosis. apCAF, which play an antigen-presenting role, can inhibit tumorigenesis (Sahai et al., 2020). In addition, the sources of CAF are again extensive. Currently, a prevailing view is that the main source of CAF is the quiescent PSC in the pancreas. However, it has also been shown that adipose-derived mesenchymal stem cells can differentiate into CAF *in vitro* and *in vivo* (Miyazaki et al., 2020), (Miyazaki et al., 2021). Moreover, bone marrow-derived macrophages also can differentiate into CAF (Iwamoto et al., 2021). Whereas the diverse source of CAF may lead to functional differences, it is necessary to study PSC as a well-defined concept.

### How Do Cancer Cells Activate PSC?

PSC are stellate stromal cells unique to the pancreas and are usually located in a quiescent state on the outer side of the acinus (Pothula et al., 2016). In addition to storing vitamin A lipid droplets, quiescent PSC also express several protein markers such as synemin and desmin (Roife et al., 2020). Accumulating evidence shows that pancreatic cancer cells can recruit and activate PSC. The activity of PSC isolated from pancreatic cancer tissue is much higher than those associated with chronic pancreatitis (Lenggenhager et al., 2019). Pancreatic cancer cells secrete a large number of paracrine cytokines, as summarized in Table 1, such as transforming growth factor-beta (TGF-*β*), Sonic hedgehog (Shh), and Interleukin-1 (IL-1) alpha to activate PSC (Sherman, 2018), while direct contact between pancreatic cancer cells and PSC, stimulating the Notch signaling pathway in PSC, leads to the activation of PSC (Fujita et al., 2009). The Notch receptors Notch1 and Notch3 are known to be highly expressed in the pancreatic cancer stroma and are accompanied by a rise in delta-like ligands Dll1, Dll3, and Dll4. Binding of the receptor to the ligand leads to the entry of the intracellular portion of the receptor into the nucleus, which activates the CSL/RBPJ protein, to promote the transcription of collagen fibers (Song and Zhang, 2018), (Procopio et al., 2015). Studies suggest that Notch3 is involved in the activation of PSC (Procopio et al., 2015). Activated PSC proliferate and secrete a large number of growth factors, inflammatory mediators, and collagen fibers that reshape the tumor microenvironment.

**TABLE 1:** PSCs activating factor secreted by cancer cells.

Cytokines	Pathway	Main function
Transforming growth factor-α (Tahara et al., 2013)	Ras-ERK, PI3K/Akt	Induces MMP-1 expression
platelet derived growth factor (Apte et al., 1999)		Increase proliferation and collagen synthesis
Galectin-1 (Masamune et al., 2006)	ERK, JNK, Activator protein-1, and NF-kappaB	Induces chemokine production and proliferation
TNF-α (Mews et al., 2002)		Increases proliferation
Shh (Khan et al., 2020)	HH	Promotes fibrosis
Plasminogen activator inhibitor-1 (Wang et al., 2019)	PAI-1/LRP-1	Promotes fibrosis
Hepatoma-derived growth factor (Chen et al., 2019)		Promotes the antiapoptosis of PSCs
Chemokine (Roy et al., 2017)		Recruits PSCs
Fibrinogen (Masamune et al., 2009)	NF-kappaB, MAPK, and ERK	Induces cytokine and collagen production
IL1β (Das et al., 2020)		Promotes immunosuppression
IL-1α (Tjomsland et al., 2011)	JAK-STAT	Promotes the secretion of inflammatory factors
TGF-β1 (Qian et al., 2010)	Smads	Promotes fibrosis
Angiotensin II (Hama et al., 2006)	Protein kinase C pathway	Promotes the proliferation
Galectin-3 (Zhao et al., 2018)	Integrin subunit beta 1 (ITGB1)	Produces inflammatory cytokines

Additionally, the rapid proliferation of cancer cells imposes significant pressure on the surroundings, including mechanical pressure which promotes the activation of PSC through a positive feedback loop (Sahai et al., 2020). G protein-coupled estrogen receptors, located within PSC membranes, sense interstitial mechanical signals and activate Ras homolog family member A (RhoA) (Cortes et al., 2019a). Both cell contraction and mechanosensation are dependent on RhoA activity, which regulates cell contractility and maintains the activated phenotype of PSC by regulating actomyosin (Cortes et al., 2019b), (Rodriguez-Hernandez et al., 2016). G protein-coupled estrogen receptor expression in the mesenchyme promotes stiffening and remodeling of the extracellular matrix (ECM), which also enhances the transmission of mechanical signals (Cortes et al., 2019c). PSC can maintain their activation state by autocrine TGF-*β* and other cytokines (Apte and Wilson, 2012). These cytokines effectively promote collagen synthesis and proliferation of PSC (Wu et al., 2020). Furthermore, pressure may be involved in the maintenance and enhancement of the activated state of PSC. Under conditions of high mechanical pressure, PSC will activate the injury-related stress response and synthesize a large number of reactive oxygen species (ROS) (Asaumi et al., 2007), which have been associated with the production of various cytokines and growth factors that facilitate the continuous activation of PSC (Richter et al., 2015). Importantly, PSC can activate the ROS system under a diverse array of conditions, including inflammation, hypoxia, and a high glucose environment, which seem to be key mechanisms for the maintenance of PSC activity (Hu et al., 2007; Lei et al., 2014; Kim et al., 2016). Furthermore, the researchers identified a key role for the Keap1-Nrf2 signalling axis in influencing the functional status of PSC in pancreatic cancer through the regulation of ROS production (Shao et al., 2019).

### The Driving Mechanism of PSC Differentiation Into Myofibroblastic and Inflammatory Subtypes

**FIGURE 1:**
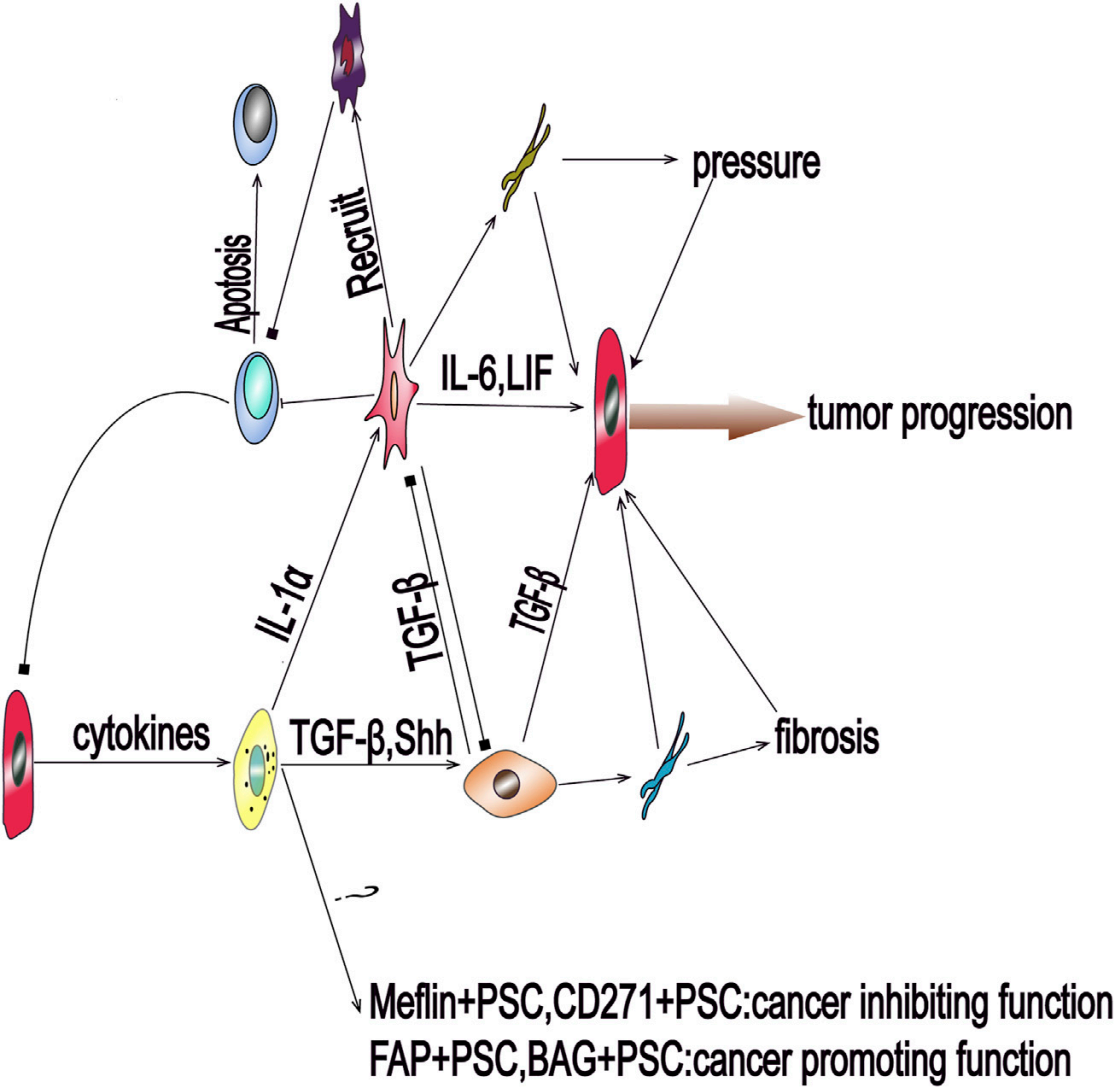
Pancreatic cancer cells can send activation signals to quiescent pancreatic stellate cells (PSCs) via paracrine factors, resulting in the activation of PSCs and differentiation into multiple subsets that participate in the progression of pancreatic cancer. Transforming growth factor beta and Sonic hedgehog can promote the activation of PSCs to myofibroblastic PSCs. The cells of this subgroup mainly secrete collagen fibers and mediate environmental fibrosis and hypoxia. Interleukin-1 alpha can promote the differentiation of PSCs into inflammatory PSCs to induce the inflammatory response and interstitial hypertension. Inflammatory PSCs also induce immunosuppression by recruiting immunosuppressive cells; BAG: Bcl2‐associated athanogene; FAP: fibroblast activation protein; IL-6: Interleukin-6; LIF: leukemia inhibitory factor; PSC: pancreatic stellate cell; Shh: Sonic hedgehog; TGF-β: transforming growth factor beta.

**FIGURE 2:**
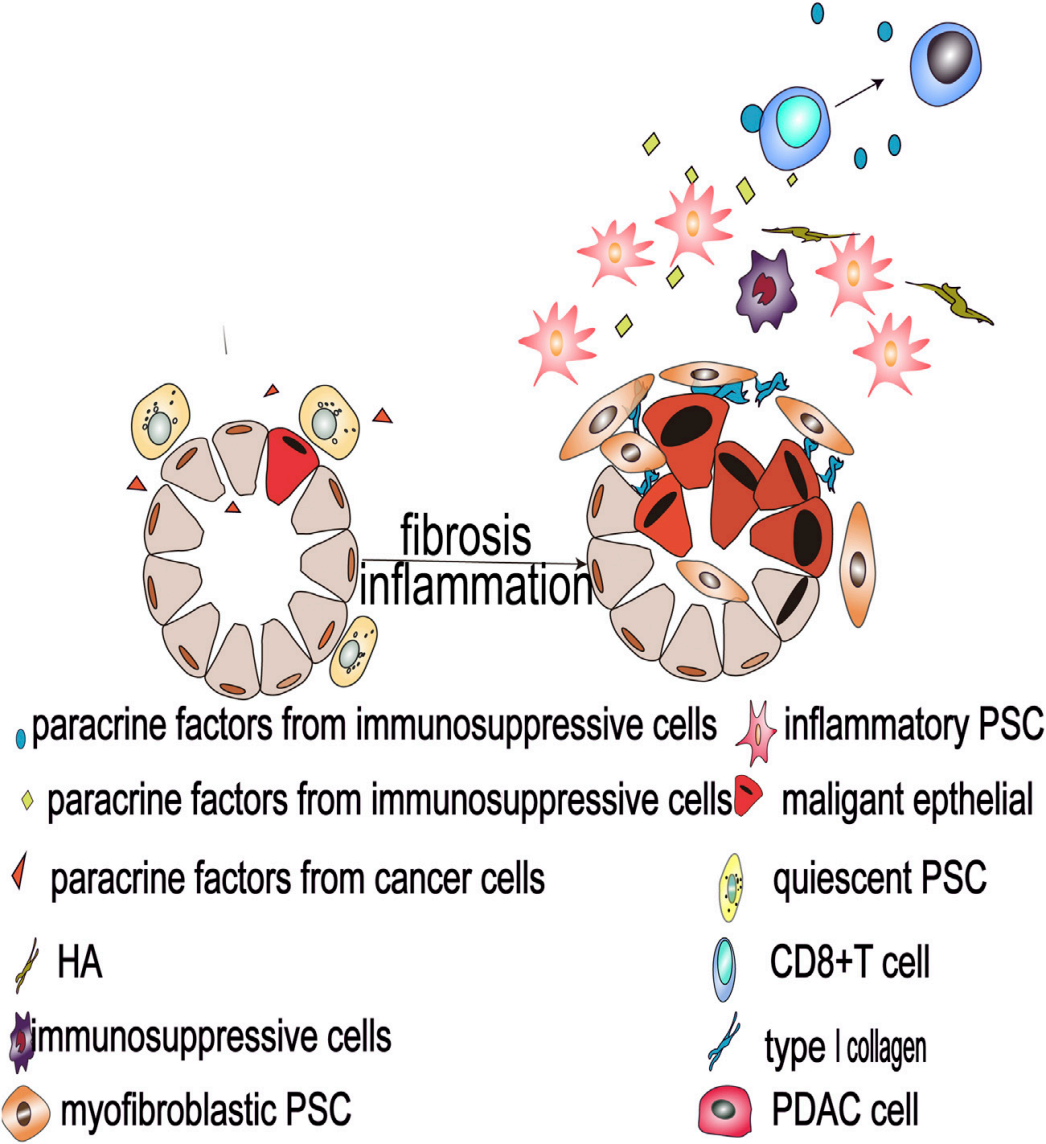
Malignant pancreatic epithelial cells activate PSCs and secrete oncogenic factors that drive PSC differentiation into myofibroblastic and inflammatory PSC subtypes, and such heterogeneous PSCs reshape the tumor microenvironment and promote the development of pancreatic cancer.

In mice and human pancreatic cancer tissues, researchers have identified two distinct subgroups of PSC. The first, myofibroblastic PSC, are associated with elevated *a*-SMA expression in immediately surrounding of cancer cells, and produce large amounts of pro-fibroproliferative collagen fibers, while the second type, inflammatory PSC, are not associated with elevated *a*-SMA expression, but are known to secrete large amounts of inflammatory cytokines further away from the cancer cells (Öhlund et al., 2017). Studies have shown that soluble cytokines secreted by cancer cells dominate this process and the differentiation of PSC into these two subtypes involves an antagonistic mechanism (Figure 1 and Figure 2). Specifically, TGF-β activates Smads signaling to drive the expression of downstream target genes, including *a*-SMA and type 1 collagen (Col1), promoting the differentiation of myofibroblastic PSC. In contrast, IL-1α activates JAK-STAT signaling to mediate mass production of Interleukin-6 (IL-6) and leukemia inhibitory factor (LIF), promoting the differentiation of inflammatory PSC (Biffi et al., 2019). IL-1α-activated PSC can assist cancer cell migration, which is known to be inhibited by TGF-β *via* blocking IL-1α-mediated secretion of hepatocyte growth factor and reducing IL-1 receptor expression (Tjomsland et al., 2016a). These factors also act antagonistically in regulating the matrix metalloproteinase profile of PSC (Tjomsland et al., 2016b). Additionally, Shh protein shows a similar regulatory effect, as it promotes the differentiation of myofibroblastic PSC and inhibits the differentiation of inflammatory PSC in the tumor microenvironment^,^ (Steele et al., 2021). Furthermore, Shh activates Hedgehog signaling, in a dose-dependent manner, to enhance the proliferation of PSC and induce the expression of *a*-SMA (Bailey et al., 2008). Moreover, the differentiation of PSC is also regulated by mechanical signal transduction, and the mechanical pressure caused by glandular dilatation is known to induce the expression of *a*-SMA in surrounding stromal cells (Miyai et al., 2020).

Indeed, whereas Öhlund identified two typical PSC subgroups, myofibroblastic PSC and inflammatory PSC, other researchers revealed the mechanism of differentiation of the two subgroups (Pothula et al., 2020), (Biffi et al., 2019). But subgroups are not stable and can re-differentiate into other types under certain conditions, including the rapid re-differentiation of inflammatory PSC into myofibroblastic PSC, in the case of monolayer distribution (Öhlund et al., 2017). This work also implies that the differentiation of PSC is dominated by cancer cells and PSC present different phenotypes according to the spatial and biochemical ecological niches in the PDA microenvironment. For myofibroblastic PSC and inflammatory PSC, the essential difference is whether *a*-SMA is expressed at high levels or not. Whether this marking method is reasonable or not needs to be further examined. Additional markers are available to differentiate diverse subgroups of PSC, such as FAP, Platelet-derived growth factor receptors, and Vimentin (Nurmik et al., 2020). It was shown that the FAP-positive PSC may produce both ECM and inflammatory cytokines (Wen et al., 2019), and the ecological niche of FAP-positive PSC in the PDA microenvironment is located between myofibroblastic PSC and inflammatory PSC (Feig et al., 2013). Thus, FAP-positive PSC may be an intermediate state between the differentiation of myofibroblastic PSC and inflammatory PSC. We need more authoritative classification criteria for the study of subgroups of PSC, which is crucial for us to further investigate the functions of PSC. Different activators, markers, and ecological niche characteristics are key factors for further identification of PSC subgroups in the future. Based on existing studies, the following section will focus on the known features and functions of myofibroblastic PSC and inflammatory PSC to further explore the functions of PSC in pancreatic cancer.

## The Function of Myofibroblastic and Inflammatory PSC

### Myofibroblastic PSC Mediate Characteristic Desmoplasia in Pancreatic Cancer

Pancreatic cancer has a high degree of desmoplasia, which is characterized by the differentiation of PSC into myofibroblastic PSC and overexpression of ECM proteins (Incio et al., 2016). Myofibroblastic PSC dominate the desmoplasia and form a physically protective barrier outside pancreatic cancer cells, protecting them from drug intervention and immune recognition, representing a significant impediment to the treatment of pancreatic cancer^,^ (Schnittert et al., 2019). This process is regulated by many myofibroblastic PSC differentiation drivers, such as TGF- *ß* and Shh. Targeting TGF-β can improve patient prognosis, whereas targeting Shh has no observable effect (Melisi et al., 2019), (Kim et al., 2014). Alternatively, the depletion of the ECM, to dissolve the fibrous barrier, was conjectured, but its application to the treatment in pancreatic cancer has not been successful. Knockout *a*-SMA transgenic mice successfully deplete *a*-SMA positive PSC in the stroma, which was associated with earlier metastasis, a high inflammatory response, and immunosuppression of pancreatic cancer (Özdemir et al., 2014), while silencing Shh in mice leads to depletion of stromal *a*-SMA^+^ cells, revealing a similar course of malignant progression (Rhim et al., 2014). Similarly, the depletion of Col1, which is the main component of the ECM, leads to immunosuppression and premature death in mice (Chen et al., 2021). Whether it is depletion of myofibroblast PSC or COL1, these are some drastic approaches. This does not directly confirm that they have a cancer-suppressive function. However, there are several possible reasons for immunosuppression and malignant progression: (Mizrahi et al., 2020): the self-protection mechanisms in pancreatic cancer cells were activated. (Kamisawa et al., 2016). the conditions created by inducing cell death in the cancer microenvironment may have primary responsibility for the adverse effects. This inspires us that targeting the mesenchymal component of pancreatic cancer requires more caution.

A novel treatment approach involves inducing PSC quiescence, which can not only effectively prevent the proliferation of connective tissue in pancreatic cancer, but also prevent the side effects caused by the depletion of the ECM. Calcipotriol, a ligand of the vitamin D receptor, is used to induce PSC quiescence and effectively reduce the degree of inflammation and fibrosis associated with pancreatic cancer in mice. The survival time of mice treated with calcipotriol chemotherapy increased by 57% (Sherman et al., 2014). All-trans retinoic acid (ATRA), an active metabolite of vitamin A, restores mechanical quiescence of PSC through a mechanism dependent on the contractile downregulation of actomyosin (Chronopoulos et al., 2016). ATRA also inhibits the ability of PSC to mechanically release active TGF-β, blocking the capacity of TGF-β to maintain the activity of PSC in an autocrine manner (Sarper et al., 2016). Recent studies have shown that ATRA-targeted stroma reduces pancreatic cancer aggressiveness (Froeling et al., 2011). A phase I clinical trial of ATRA-induced PSC quiescence demonstrates that patients tolerate ATRA well (Kocher et al., 2020); however, further clinical trials are required to verify the effectiveness of ATRA.

As the depletion of *a*-SMA positive stromal cells leads to the malignant progression of pancreatic cancer, an important question arises as to whether myofibroblastic PSC can inhibit tumor progression. This is likely not the case, and the expression of *a*-SMA in pancreatic cancer tissues is unstable and provides no predictive value in the prognosis of patients (Erkan et al., 2008; Moffitt et al., 2015; Haeberle et al., 2018). Studies have shown that the depletion of myofibroblastic PSC will lead to a substantial expansion of the number of inflammatory PSC, which then become the dominant subgroup in the tumor microenvironment, leading to the malignant progression of pancreatic cancer. This implies that an increase in the number of inflammatory PSC may be the main reason for the deterioration of *a*-SMA^-^ mice (Steele et al., 2021). In addition, Col1 plays an important role in sending malignant signals to cancer cells (Grzesiak et al., 2007). Studies show that patients with low Col1 have a median survival time of 14.6 months, in comparison to 6.4 months for patients with high levels (Whatcott et al., 2015). *α*1*β*2 integrin is an *α/β* heterodimer membrane protein and the main receptor mediating the adhesion of cancer cells to the surrounding Col1 (Naci et al., 2015). Once these two elements combine to form adhesion plaques on the cell surface, they provide an important medium for the ECM to transmit signals into the cell, activating downstream Src family kinases, focal adhesion kinase, and extracellular regulated kinase signaling pathways (Ivaska and Heino, 2011), (Cooper and Giancotti, 2019). Discoidin domain receptor 1 (DDR1) is a member of the receptor tyrosine kinase family and acts as an important receptor for Col1 (Baltes et al., 2020). DDR1 is widely expressed in epithelial cells, involved in the regulation of multiple signaling pathways, and is known to have a strong association with cancer progression (Henriet et al., 2018). Col1 can transmit a variety of carcinogenic signals to cells, through the two pathways described above, to promote the migration, proliferation, and drug resistance of pancreatic cancer.

**FIGURE 3:**
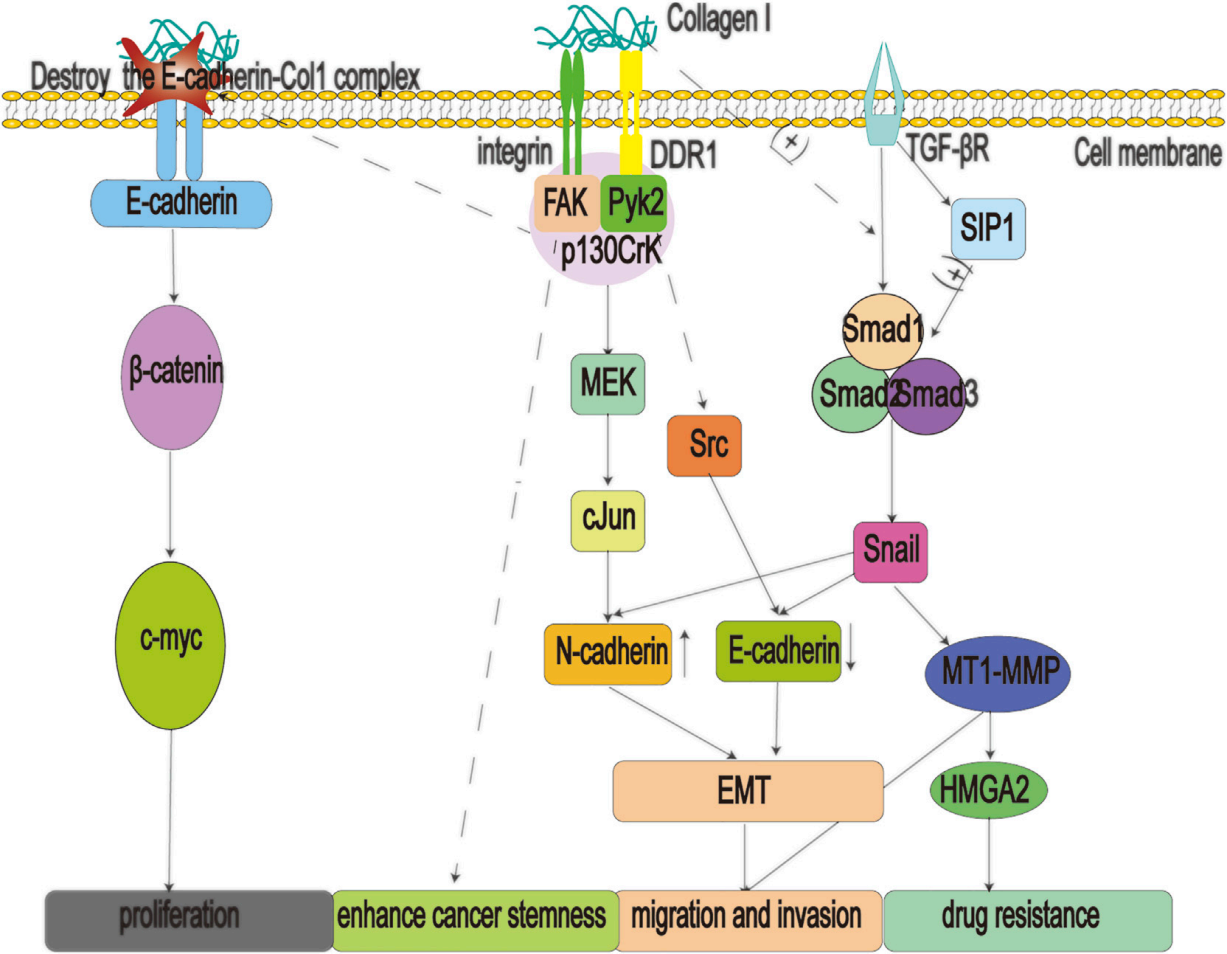
Type 1 collagen (Col1) fibers are secreted by myofibroblastic pancreatic stellate cells (PSCs), which can promote multiple processes such as migration, proliferation, drug resistance, and stemness enhancement of pancreatic cancer cells, by binding to *α*1*β*2 integrins and discoidin domain receptors 1 (DDR1). In addition, when the E-cadherin-Col1 complex is destroyed, it will lead to the accumulation of *ß*-catenin in the nucleus, activate the oncogene c-myc, and eventually lead to the proliferation of pancreatic cancer cells; Col1: type 1 collagen; DDR1: Discoidin domain receptors one; EMT: epithelial mesenchymal transition; FAK: focal adhesion kinase; MET: metformin; HMGA2: high mobility group A2; MMPs: matrix metalloproteinases; MT1-MMP: membrane-type matrix metalloproteinase-1; SIP1: smad interacting protein one; TGF-βR: transforming growth factor beta receptor.

Col1 makes an outstanding contribution in promoting the malignant procession of cancer (Figure 3). Col1 signals the p130Crk-associated substrate and activates the JNK signaling pathway to increase N-cadherin expression (Huang et al., 2016), (Shintani et al., 2006) DDR1 also activates downstream Src to decrease the expression of E-cadherin (Sun et al., 2008), (Chen et al., 2016). These factors reduce the adhesion of cancer cells to the adjacent extracellular matrix and help them to complete their EMT, greatly enhancing their ability to migrate. In addition, Col1 can engage in crosstalk with transforming growth factor-beta (TGF-β) signaling to promote EMT in cancer cells. Studies have shown that Col1 increases the expression of transcription factor Snail by interacting with TGF-β-Smads. Moreover, the up-regulation of Snail can not only promote the occurrence of EMT but also increase the expression of membrane-type matrix metalloproteinase-1, which further helps pancreatic cancer cells to dissolve collagen fibers and achieve metastasis (Shields et al., 2011). Furthermore, Col1 positively regulates the transcription of Snail via the Smad interacting protein 1, a positive regulator of Smads signal (Imamichi et al., 2007). Col1 also induces the proliferation of pancreatic cancer cells by disrupting the E-cadherin-Col1 complex, leading to the accumulation of *ß*-catenin in the nucleus and activating the transcription of the oncogene c-Myc (Koenig et al., 2006; Katoh, 2018). Overexpression of c-Myc promotes the immortalization of cancer cells (Dang, 2012) and alters the original signal transduction mode of cells, resulting in malignant cell proliferation, anti-apoptosis, and chromosome instability (Kolenda et al., 2020). Moreover, the *α*1*β*2 integrin pathway can also assist cells to acquire a cancer stem cell phenotype (Begum et al., 2017). Finally, Col1 mediates drug resistance by activating MT1-MMP to increase the expression of high mobility group A2, a non-histone DNA-binding nuclear protein involved in chromatin remodeling and gene transcription (Dangi-Garimella et al., 2011).

### Interstitial Hypertension Dominated by Inflammatory PSC Is a Natural Protective Barrier for Pancreatic Cancer

Hyaluronic acid (HA) is a linear glycosaminoglycan macromolecule composed of repeating units that are synthesized by hyaluronan synthase enzymes HAS1, HAS2, and HAS3, and is the main source of external pressure in the cancer stroma. HA can also specifically bind to the CD44 protein to affect the physiological activity of cancer cells (Sato et al., 2016; Caon et al., 2020). Studies have shown that the content of HA in pancreatic cancer is higher than in other cancer tissues, which leads to the characteristic interstitial hypertension observed in pancreatic cancer (Jacobetz et al., 2013). The content of HA in pancreatic cancer is 12 times higher than that in a healthy pancreas (Theocharis et al., 2000). Studies show that activated PSCs are the main source of HA (Junliang et al., 2019). Surprisingly, HA is not synthesized by myofibroblastic PSC, and their depletion does not affect the expression of HA in pancreatic cancer stroma (Özdemir et al., 2014). In contrast, inflammatory PSC express HAS1 and HAS2, suggesting that these cells represent the main source of HA (Elyada et al., 2019). Pancreatic cancer has extremely high external pressure in the interstitium, which contributes to difficulties associated with treatment (DuFort et al., 2016). HA-induced external pressure forms a physical barrier to help pancreatic cancer cells resist the effects of therapeutic intervention (Jacobetz et al., 2013). Studies have shown that low molecular weight HA enhances the migration of cancer cells (Junliang et al., 2019), whereas high molecular weight HA is the main cause of external pressure, leading to interstitial pressures of up to 100 mmHg, resulting in vascular collapse, impeding the delivery of nutrients, oxygen, and drugs, and reducing the infiltration of immune cells (Chauhan et al., 2014). A recent study shows a median survival time of 24.3 months for patients with low levels of HA, in comparison to 9.3 months for patients with high levels (Whatcott et al., 2015). The degradation of HA in pancreatic cancer tissue by administering halofuginone can significantly weaken the effect of the physical barrier, resulting in prolonged survival time in mice (Elahi-Gedwillo et al., 2019).

Dissolving HA with drugs represents a viable treatment option for pancreatic cancer patients (Sato et al., 2016). Compounds capable of dissolving HA in pancreatic tumor-bearing mice include 4-methylumbelliferone (Nagy et al., 2015), Minnelide (Banerjee et al., 2016), and PEGylated human recombinant hyaluronidase (PEGPH20) (Hingorani et al., 2016). PEGPH20 has been shown to significantly prolong disease-free survival of pancreatic cancer patients, especially in patients with high HA expression. Maximum survival was observed upon treatment with a combination therapy regimen including PEGPH20, albumin-bound paclitaxel, and gemcitabine (Hingorani et al., 2018; Ramanathan et al., 2019). Unfortunately, clinical trials of PEGPH20 were eventually discontinued due to pharmacological toxicity.

Several pancreatic cancer cell lines with HA receptor expression exhibit potential for hypo-differentiation and high migration (Abetamann et al., 1996; Sugahara et al., 2008). Research has shown that high levels of HA can lead to the malignant progression of pancreatic cancer. HA binding to the CD44 receptor mediates cancer cell EMT, drug resistance, and proliferation through both RAS/ERK and PI3K/AKT signaling pathway activation (Sato et al., 2016). Recently, researchers have developed a therapeutic approach involving a combination of HA and CD44 targeting. Specifically, the drug is modified by HA and targeted to CD44-positive tumor cells to improve the efficiency of drug utilization (Mattheolabakis et al., 2015). Many preclinical trials have revealed positive results using HA-modified drugs, as shown in Table 2.

**TABLE 2:** Targeting CD44-positive pancreatic cancer cells with HA-modified drugs.

Drug	Auxiliary materials	Synthetic drug	Mechanism
3,4-difluorobenzylidene curcumin (CDF) (Kesharwani et al., 2015a)	Poly (amidoamine) (PAMAM)	HA-PAMAM-CDF	Inhibits NF-κB signaling and reduces CD44 expression
CDF (Kesharwani et al., 2015b)	styrene maleic acid (SMA)	HA-SMA-CDF	Inhibits NF-κB signaling and reduces CD44 expression
Gemcitabine and quercetin (Serri et al., 2019)	nanoparticles	Gemcitabine and quercetin encapsulated in HA modified nanoparticles	Anti-inflammatory effect and metabolic intervention of DNA
Cu(DDC)_2_ (Marengo et al., 2019)	Liposome	Encapsulation of Cu (DDC) 2 complex in HA modified liposomes	ROS-mediated anticancer activity
Gemcitabine (Dalla Pozza et al., 2013)	Liposome	Gemcitabine complex encapsulated in HA modified liposomes	Interferes with DNA synthesis
Drugs (Wei et al., 2013)	nanogels	The drug encapsulated in HA modified nanogels	
5-FU (Nigam Joshi et al., 2017)	Ag-GQDs	5-FU encapsulated in HA modified Ag-GQDs	Anti-tumor proliferation
metformin (MET) (Farag et al., 2021)	Metformin-Phospholipid Sonocomplex (MPS)	HA-MPS-MET	Corrects microenvironment hypoxia

### Inflammatory PSC Lead to Malignant Inflammation of Pancreatic Cancer

**FIGURE 4:**
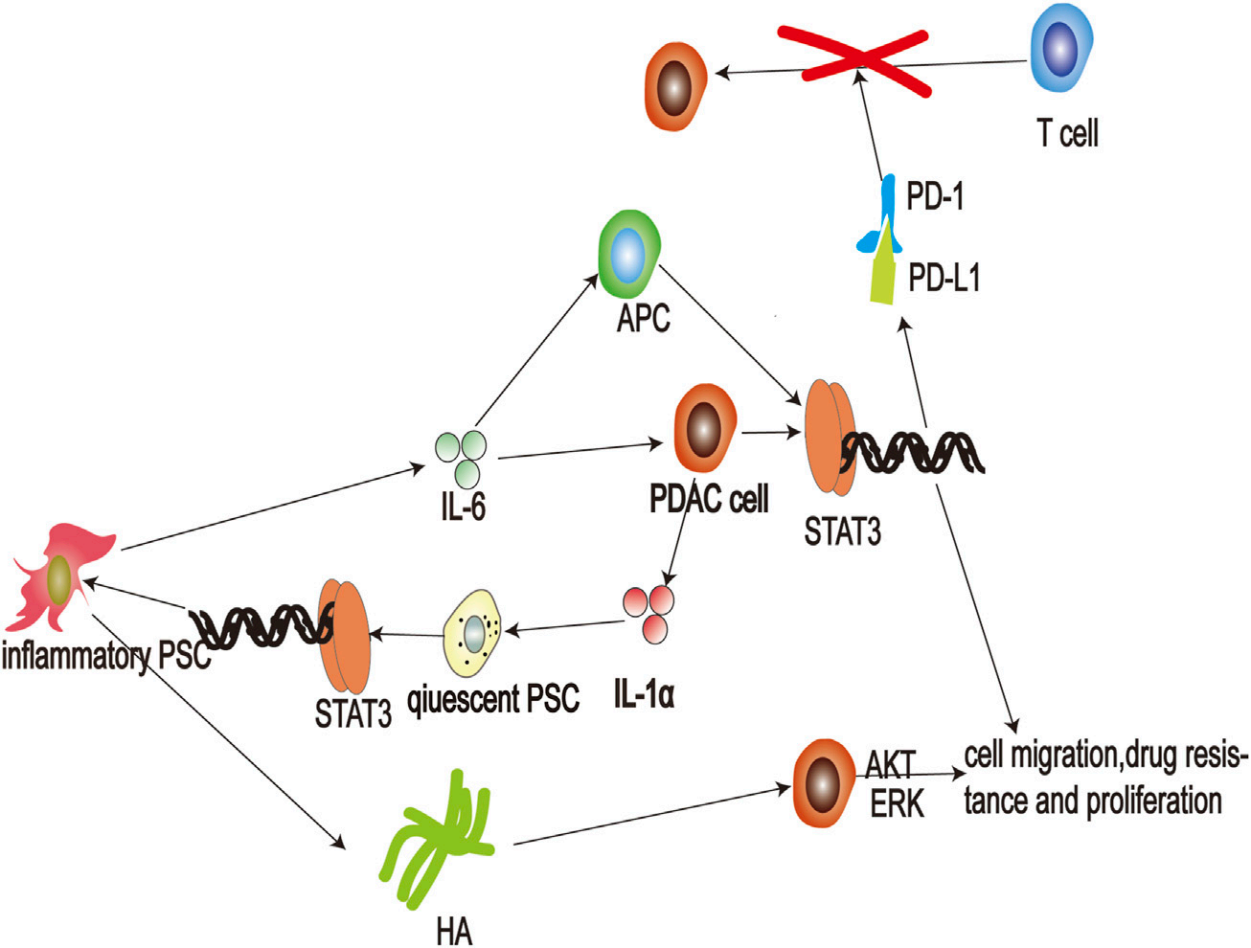
Schematic representation of the interaction between pancreatic cancer cells and inflammatory pancreatic stellate cells (PSCs). Secretion of interleukin (IL)-1*α* by pancreatic cancer cells stimulates activation of JAK-STAT3 signaling in quiescent PSCs, and leads to differentiation of inflammatory PSCs. Thereafter, inflammatory PSCs produce hyaluronic acid that then binds to cluster of differentiation (CD) 44 to activate AKT and ERK signaling pathways and promote the malignant process of pancreatic cancer. Additionally, inflammatory PSCs can also secrete large amounts of the cytokine IL-6, which activates JAK-STAT3 signaling pathways in cancer cells, promoting their proliferation and invasion. In addition, IL-6 also promotes the transcription of PD-L1 in antigen-presenting cells and cancer cells, leading to suppression of T-cell immunity; APC: antigen-presenting cell; ERK: extracellular regulated kinase; HA: hyaluronic acid; HAS: hyaluronan synthase; IL-1: Interleukin-1 IL-6: Interleukin-6; PD-1: programmed cell death one; PD-L1: protein programmed cell death one ligand one; PSC: pancreatic stellate cell.

Inflammation in the tumor microenvironment assists in the process of drug resistance, proliferation, metastasis, and immunosuppression in pancreatic cancer (Jarrin Jara et al., 2020). However, IL-6, secreted by inflammatory PSC, plays a significant role in mediating inflammation-related malignant progression of pancreatic cancer (Figure 4). Research has shown that IL-6 expression levels are significantly higher in patients with systemic metastases, and high levels of IL-6 reliably predict poor prognosis in patients treated with surgery (Palmquist et al., 2020). Additionally, high levels of IL-6 are often accompanied by large tumor size and distant metastases (Miura et al., 2015). IL-6 activates the JAK-STAT3 signaling pathway in cancer cells to exert its carcinogenic effect (Nagathihalli et al., 2016) and is regulated by both classical and trans-signaling pathways. In classical signaling, IL-6 can bind to the IL-6 receptor and induce a conformational change that triggers glycoprotein (gp) 130 dimerization. Subsequently, two IL-6-IL-R molecules bind to a gp130 dimer forming a 6-membered complex resulting in the activation of downstream JAK (Yu et al., 2014). In trans-signaling, the cleavage of IL-6R by specific enzymes, or alternative splicing of IL6R mRNA, produces soluble IL-6R (sIL-6R) (Johnson et al., 2018). IL-6R binds sIL-6R and forms an L-6-sIL-6R complex with the ability to induce gp130 dimerization to activate downstream JAK (104). Both types of regulation result in JAK activation and phosphorylation of the transcription factor STAT3, which regulates gene expression in cancer cells (van Duijneveldt et al., 19792020). Studies have shown that activating the JAK-STAT3 pathway enhances migration (Okitsu et al., 2010), drug resistance, proliferation (Zhang et al., 2017), and stemness (Alcalá et al., 2019) of pancreatic cancer cells. Inhibition of STAT3 signaling leads to apoptosis of pancreatic cancer cells (Nagathihalli et al., 2015). IL-6-receptor blockers enhance chemotherapeutic efficacy in KPC (LSL-Kras G12D/+; LSL-Trp53 R172H/+; Pdx-1Cre) mice (Long et al., 2017).

Furthermore, inflammatory PSC also secrete IL-6 resulting in immunosuppression. PD-1 is one of the co-suppressor receptors of activated immune cells, and PD-L1 is mainly expressed in tumor cells and antigen-presenting cells (Ai et al., 2020). When PD-L1 binds to PD-1, this leads to suppression of activated immune cells and assists in the immune escape of tumor cells (Patel and Kurzrock, 2015). Anti-PD-1 targeted therapy has been successfully used in many cancers, but not in pancreatic cancer (Tsoukalas et al., 2019). This may be due to the persistent inflammatory response in pancreatic cancer (Antonangeli et al., 2020). Research has shown that transcription of PD-L1 is regulated by IL-6 in pancreatic cancer (Tsukamoto et al., 2018). IL-6 accelerates bone marrow-derived myeloid-derived suppressor cell differentiation and leads to increased levels of PD-L1 expression on myeloid-derived suppressor cells (Marigo et al., 2010; Weber et al., 2021). IL-6 also induces apoptosis of conventional type 1 dendritic cells to prevent antigen presentation (Lin et al., 2020). Moreover, antibody blockade of IL-6 reduces the expression of PD-L1 in dendritic cells (Eriksson et al., 2019). JAK-STAT3 activation in pancreatic tumor-bearing mice inhibits the effect of anti-PD-1 treatment (Lu et al., 2017). Therefore, blocking IL-6 may represent an effective adjuvant method for anti-PD-1 therapy. The combination of anti-IL-6 and anti-PD-L1 therapy effectively increases the survival rates of pancreatic cancer mice (Mace et al., 2018).

Studies focused on blocking IL-6-mediated JAK-STAT3 signaling activation have been conducted in the following categories ((Kaur et al., 2020): (Mizrahi et al., 2020) IL-6 receptor antagonists: tocilizumab, sarilumab; (Kamisawa et al., 2016); IL-6 production inhibitors: olokizumab, sirukumab, siltuximab, clazakizumab, PF-423691; and (Wang et al., 2020) JAK1/2 and STAT3 inhibitors: ruxolitinib, momelotinib. Despite the success of blocking IL-6 in pancreatic cancer animal models, this approach was virtually ineffective in a clinical setting (Ng et al., 2019). Anti-IL-6 therapy as an adjuvant in combination with other targeted therapies may represent a novel area worthy of exploration in the treatment of pancreatic cancer.

Outlook: The diversity of PSC subsets represents an important factor in the therapeutic intervention of pancreatic cancer.

We have shown that different groups of PSC play different roles in the progression of pancreatic cancer. The discovery of PSC heterogeneity will provide the basis for the development of novel approaches for the treatment of pancreatic cancer. Finding therapeutic targets for the treatment of pancreatic cancer at the level of PSC heterogeneity represents an unexplored potential therapeutic strategy. Although most of our regimens targeting myofibroblastic PSC for pancreatic cancer (including depletion of *a*-SMA + PSC and blockade of Shh) have failed, we have still achieved some successes such as blocking TGF-β or inducing PSC quiescence. Simultaneous inhibition of inflammatory responses and fibroplasia caused by different PSC subgroups significantly prevented pancreatic cancer progression in mice with no side effects (Khan et al., 2020). These reveal that targeting PSC for pancreatic cancer is an extremely valuable idea, but it should be done rationally. Future research should focus on the following aspects: 1) Induction of PSC quiescence to effectively block the progression of pancreatic cancer, 2) Evaluation of how pancreatic cancer cells produce and regulate the production of PSC subsets, and how PSC tumor suppressor subsets, specifically CD271 + PSC (Nielsen et al., 2020) and Meflin + PSC (Mizutani et al., 2019), are formed, and 3) Simultaneous blockade of the oncogenic effects of different PSC subgroups to treat pancreatic cancer more effectively.

## Glossary

**ATRA:**: All-trans retinoic acid
CAF: Cancer-associated fibroblasts
**Coll:**: type 1 collagen.
**DDR1:**: Discoidin domain receptors one
**ECM:**: extracellular matrix.
**FAP:**: fibroblast activation protein
**HA:**: hyaluronic acid.
**HAS:**: hyaluronan synthase.
**IL-1:**: Interleukin-1.
**IL-6:**: Interleukin-6.
**IL-6R:**: Interleukin-6 receptor.
**LIF:**: leukemia inhibitory factor.
**PD-1:**: programmed death-ligand 1.
**PD-L1:**: protein programmed cell death one ligand 1.
**PEGPH20:**: PEGylated human recombinant hyaluronidase.
**PDGF:**: platelet derived growth factor.
**PSC:**: pancreatic stellate cell.
**a-SMA:**: a-smooth muscle actin.
**RhoA:**: Ras homolog family member A
**ROS:**: reactive oxygen species
**Shh:**: Sonic hedgehog.
**TGF-β:**: transforming growth factor beta
